# Antipsychotic Treatment of Behavioral and Psychological Symptoms of Dementia (BPSD): Management of Extrapyramidal Side Effects

**DOI:** 10.3389/fphar.2019.01045

**Published:** 2019-09-17

**Authors:** Yukihiro Ohno, Naofumi Kunisawa, Saki Shimizu

**Affiliations:** Department of Pharmacology, Osaka University of Pharmaceutical Sciences, Takatsuki, Japan

**Keywords:** behavioral and psychological symptoms of dementia (BPSD), extrapyramidal side effects (EPS), antipsychotics, anti-Alzheimer’s disease drugs, antidepressants, 5-HT receptors

## Abstract

Antipsychotic drugs are often used for the treatment of behavioral and psychological symptoms of dementia (BPSD), especially psychosis and behavioral disturbances (e.g., aggression and agitation). They are prescribed alone or in conjunction with anti-dementia (e.g., anti-Alzheimer’s disease drugs) and other psychotropic drugs (e.g., antidepressants). However, antipsychotic drugs frequently produce serious extrapyramidal side effects (EPS) including Parkinsonian symptoms (e.g., bradykinesia, akinesia, tremor, and muscle rigidity). Therefore, appropriate drug choice and combination strategy are important in the treatment of BPSD. Among anti-Alzheimer’s disease drugs, cholinesterase inhibitors (ChEIs, e.g., donepezil and galantamine) have a propensity to potentiate EPS associated with antipsychotic treatment in a synergistic manner. In contrast, the NMDA receptor antagonist memantine reduces antipsychotic-induced EPS. Antidepressant drugs, which inhibit 5-HT reuptake into the nerve terminals, also synergistically augment antipsychotic-induced EPS, while mirtazapine (α_2_, 5-HT_2_ and 5-HT_3_ antagonist) reduces the EPS induction. Importantly, previous studies showed that multiple 5-HT receptors play crucial roles in modulating EPS associated with antipsychotic treatment. Specifically, activation of 5-HT_1A_ receptors or blockade of 5-HT_2_, 5-HT_3_ and 5-HT_6_ receptors can alleviate EPS induction both by antipsychotics alone and by combined antipsychotic treatments with ChEIs or 5-HT reuptake inhibitors. In this article, we review antipsychotic use in treating BPSD and discuss the favorable drug selection in terms of the management of antipsychotic-induced EPS.

## Introduction

Dementia is a neurodegenerative brain disorder with diverse clinical symptoms including cognitive impairment (e.g., memory loss and learning deficits) and non-cognitive disorders (e.g., behavioral and psychological deficits). Nearly 50 million patients worldwide develop dementia and this population is expected to exceed 130 million in 2050 (Prince et al., 2015; Jin and Liu, 2019). The global cost associated with dementia was about 1,000 billion dollars in 2015, and this continues to increase rapidly. There are numerous causes of dementia including Alzheimer’s disease, cerebrovascular diseases, Parkinson’s disease, Lewy body disease, and mixed types, among which Alzheimer’s disease is the most frequent (Lee et al., 2004; Prince et al., 2015; Sturm et al., 2018).

**Figure 1:**
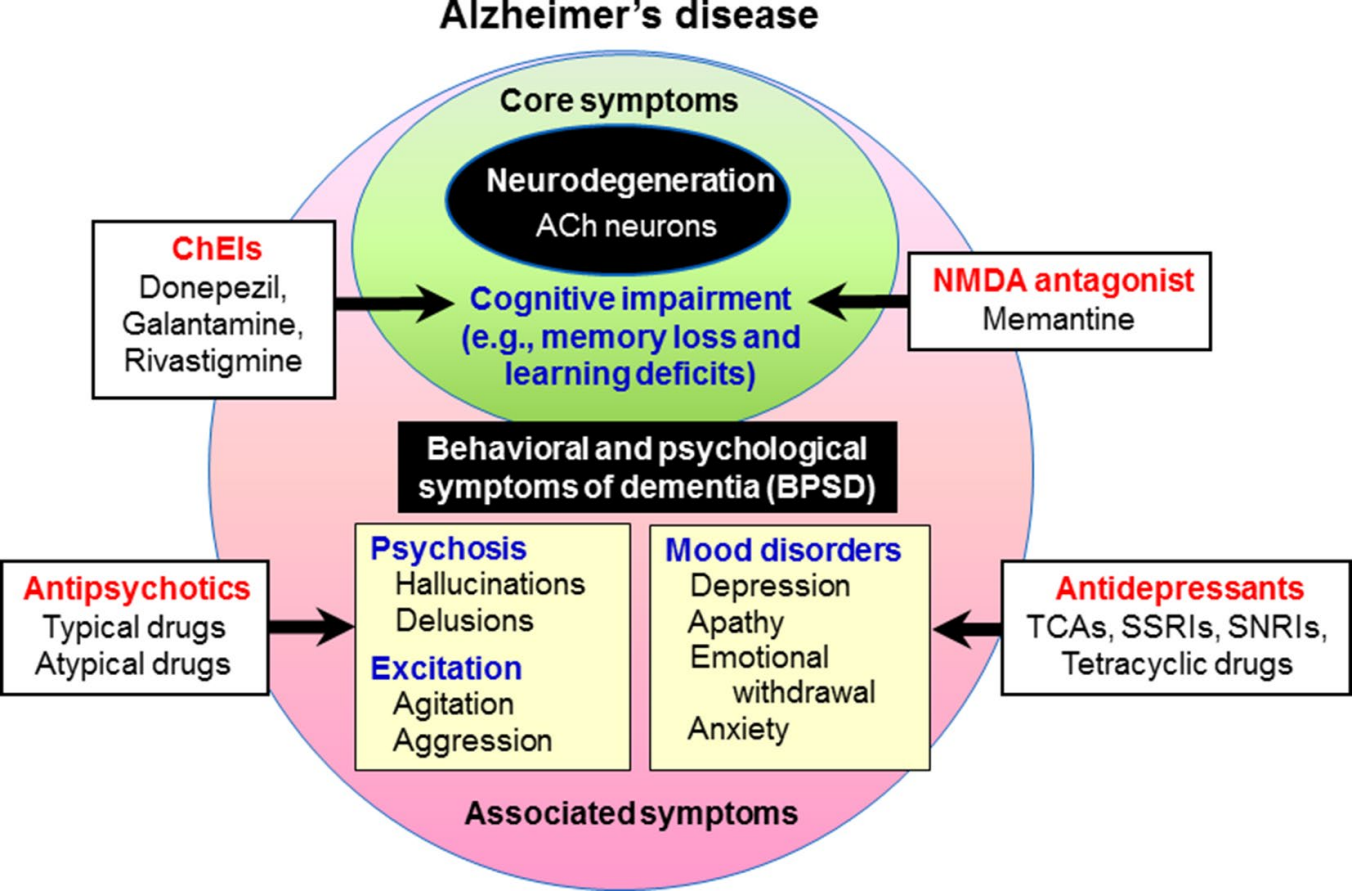
Behavioral and psychological symptoms of dementia (BPSD) in Alzheimer’s disease. Patients with dementia show not only core symptoms of dementia (e.g., memory loss and learning deficits), but also various associated symptoms including BPSD (e.g., psychosis, behavioral excitation, and mood disorders). Cognitive impairment in Alzheimer’s disease is usually treated with cognitive enhancers such as the cholinesterase inhibitors (ChEIs, e.g., donepezil, galantamine, and rivastigmine) and the NMDA antagonist (e.g., memantine). In the treatment of BPSD, antipsychotic drugs are used for psychosis and behavioral disturbances, and antidepressants for depressive mood.

Behavioral and psychological symptoms of dementia (BPSD) occur in the majority (up to 90%) of dementia patients, and this causes significant distress to both patients and caretakers (O’Donnell et al., 1992; O’Brien, 2003; Rosdinom et al., 2013). BPSD includes behavioral excitement (e.g., agitation and aggression), mood disorders (e.g., apathy, depression and anxiety), psychosis (e.g., hallucinations and delusions) and other symptoms (e.g., eating disturbances and sleep disorders) (**Figure 1**). Although the prevalence of BPSD varies among reported studies, hallucinations occur in 15–50% of patients with dementia, delusions in 10–75% and behavioral disturbances (e.g., agitation and aggression) in about 50%, while affective symptoms are less common (van der Linde et al., 2014; Devshi et al., 2015). To treat BPSD, non-pharmacological interventions such as cognitive stimulation training, exercise, music therapy, light therapy and aromatherapy are recommended as first-line treatments. Nonetheless, pharmacological treatments with antipsychotics and other psychotropic drugs are necessary to treat BPSD (Brimelow et al., 2019; Jin and Liu, 2019; Kales et al., 2019) (**Figure 1**). Specifically, antipsychotic drugs are the first choice to reduce psychosis and behavioral disturbances despite their frequent side effects (Lee et al., 2004; Trifirò et al., 2009; Brimelow et al., 2019; Sturm et al., 2018).

It is well known that antipsychotic drugs commonly cause serious extrapyramidal side effects (EPS) (e.g., bradykinesia, muscle rigidity, tremor, and akathisia) by blocking dopamine D_2_ receptors in the striatum (Remington and Kapur, 1999; Kapur and Remington, 2001; Ohno et al., 2013; Ohno et al., 2015; Ohno, 2019). Antipsychotic-induced EPS often leads to suboptimal treatment of BPSD or treatment discontinuation. In addition, recent studies showed that cholinesterase inhibitors (ChEIs), licensed drugs for cognitive impairment due to Alzheimer’s disease, potentiate EPS induction with antipsychotic treatments (Shimizu et al., 2015). It is therefore important to understand the mechanism underlying antipsychotics-induced EPS and antipsychotic drug interactions with other medications in the treatment of BPSD.

In this article, we review the pharmacological features of antipsychotic drugs, especially those related to EPS, and discuss the proper usage and selection of antipsychotics in treating BPSD in terms of EPS management.

## Antipsychotic Use in BPSD Treatment

Antipsychotic drugs are used to treat BPSD with a prescription rate of about 20–50% (Lee et al., 2004; Brimelow et al., 2019; Sturm et al., 2018). The target symptoms of antipsychotic drugs include agitation, aggression, psychosis, and inappropriate behaviors (**Figure 1**). None of the antipsychotics, except for haloperidol and risperidone in several countries, are approved to treat BPSD; therefore, these drugs are generally prescribed as off-label. Nonetheless, antipsychotic drugs are reported to produce significantly better improvements than placebos in treating BPSD (Lee et al., 2004; Brimelow et al., 2019; Sturm et al., 2018).

**Figure 2:**
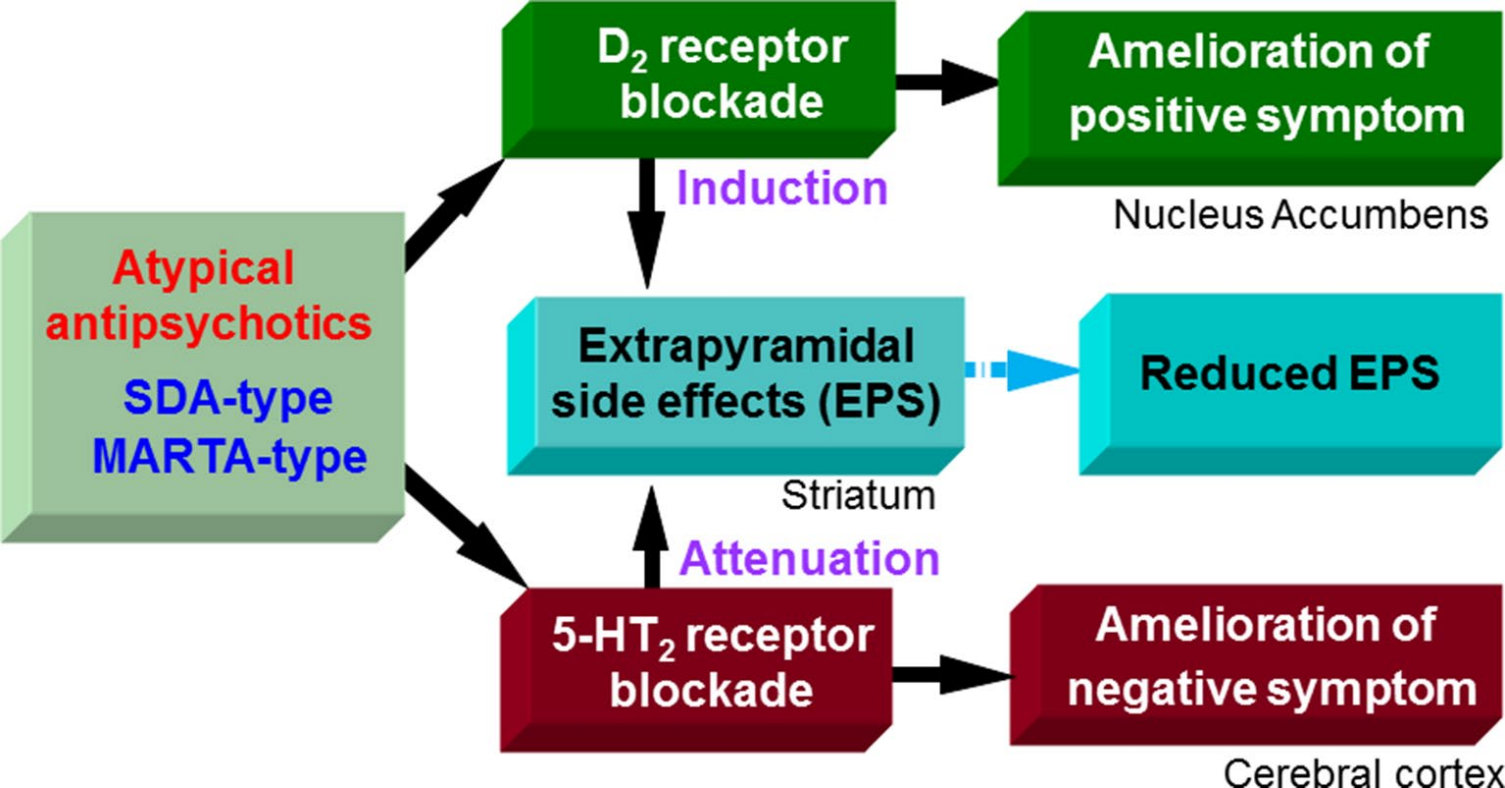
Pharmacological actions of atypical antipsychotics. Likely typical antipsychotic drugs, D_2_ blocking actions of serotonin and dopamine antagonists (SDA)-type and multiple-acting receptor targeted antipsychotics (MARTA)-type antipsychotics ameliorate positive symptoms (e.g., hallucination, delusions, and excitation) in schizophrenia, but induce extrapyramidal side effects (EPS). On the other hand, SDA-type and MARTA-type antipsychotics show higher 5-HT_2_ than D_2_ binding affinities and possess potent 5-HT_2_ antagonistic actions. The 5-HT_2_ blocking activities of SDA-type antipsychotics ameliorate negative symptoms (e.g., apathy and social withdrawal) in schizophrenia and can reduce EPS. Thereby, overall EPS liability of SDA-type antipsychotics is lower than typical antipsychotics (D_2_ antagonists).

Antipsychotic drugs commonly possess dopamine D_2_ blocking actions. It is known that D_2_ receptor blockade by antipsychotics in the cortico-limbic regions (e.g., nucleus accumbens) contributes to antipsychotic activities, which alleviates psychosis (e.g., hallucinations and delusions) and behavioral excitation (e.g., agitation, aggression and hyperactivity) (**Figure 2**). However, it should be noted that all antipsychotic drugs frequently cause extrapyramidal motor disorders due to the striatal D_2_ receptor blockade, which disrupts the effective treatment of BPSD.

**Figure 3:**
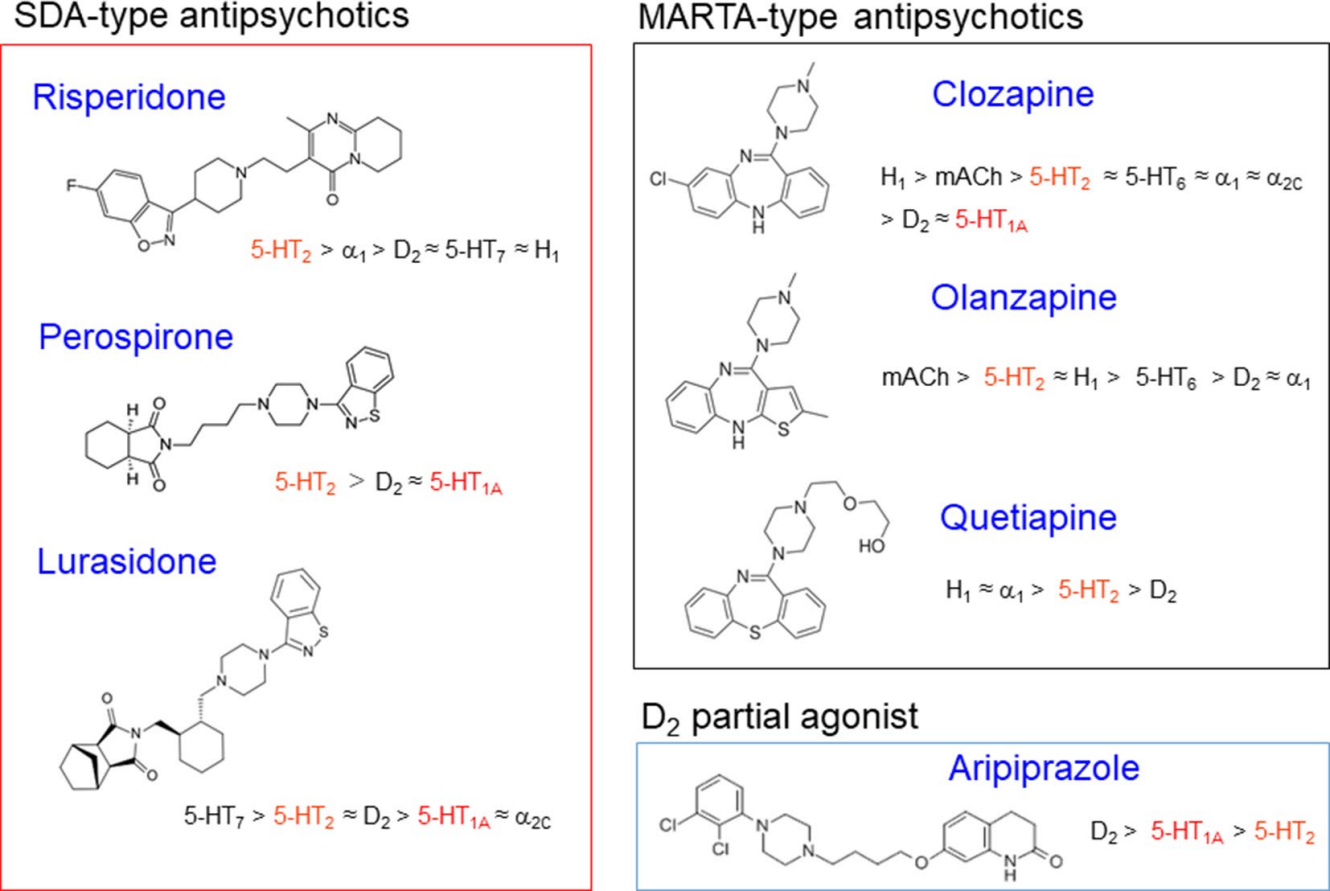
Classification and characteristics of atypical antipsychotic drugs. Figure shows chemical structures of atypical antipsychotics with their receptor binding profiles (affinities). Most atypical antipsychotics possess potent 5-HT_2_ blocking actions and act as serotonin and dopamine antagonists (SDAs). Among SDAs, clozapine derivatives (e.g., olanzapine and quetiapine) show various actions on other receptors than D_2_ and 5-HT_2_ receptors, including histamine H_1_, adrenergic α_1,_ and muscarinic blocking actions. Thereby, they are sometimes called as multi-acting receptor-targeted antipsychotics (MARTAs) and differentiated from SDAs (e.g., risperidone, perospirone, and lurasidone). Although aripiprazole possess moderate 5-HT_2_ blocking activities, it primarily acts as a dopamine D_2_ partial agonist. Furthermore, several atypical antipsychotics have own characteristics such as 5-HT_1A_ partial agonistic actions for perospirone, lurasidone and aripiprazole, 5-HT_6_ blocking actions for olanzapine and quetiapine, and 5-HT_3_ blocking actions for olanzapine.

Antipsychotic drugs are generally classified into two groups, typical and atypical (Ohno et al., 1997; Ohno et al., 2012). Typical antipsychotics are the classic standard drugs and frequently cause severe EPS. Based on their chemical structures, they are grouped into several classes, phenothiazines (e.g., chlorpromazine and fluphenazine), butyrophenones (e.g., haloperidol and spiperone), benzamides (e.g., sulpiride and tiapride), and others. On the other hand, atypical antipsychotics were developed as second generation, and are generally less potent than typical ones in inducing EPS (**Figures 2** and **3**). These include the serotonin and dopamine antagonists (SDAs) with potent blocking action for 5-HT_2_ receptors, the multiple-acting receptor targeted antipsychotics (MARTAs) and the dopamine D_2_ partial agonists (Ohno et al., 2012). Besides reduced EPS, these drugs were originally expected be superior to typical antipsychotics in terms of their efficacy to treat negative symptoms (e.g., apathy and emotional withdrawal) (**Figure 2**). However, comprehensive clinical studies including the Clinical Antipsychotic Trials of Intervention Effectiveness (CATIE) and European First-Episode Schizophrenia Trial (EUFEST), revealed no clear advantages of atypical over typical drugs in terms of efficacy (Lieberman et al., 2005; Keefe et al., 2007; Davidson et al., 2009). Nonetheless, due to the reduced side effect profile, atypical antipsychotics are widely used as a first line drug in BPSD treatment as well as schizophrenia treatment.

## Antipsychotic-Induced EPS

### Clinical Symptoms

Major EPS symptoms associated with antipsychotic treatment of BPSD include Parkinsonian symptoms, akathisia, and dystonia. Tardive dyskinesia (repeated abnormal involuntary movements) is another antipsychotic-induced EPS, but is rare during the relatively short-term BPSD treatment as it is a chronic side effect associated with long-term antipsychotic treatment and usually appears upon the cessation of treatment.

### Parkinsonian Symptoms

Antipsychotic-induced Parkinsonian symptoms are involuntary movement disorders including bradykinesia, tremor and muscle rigidity (Samii et al., 2004; Haddad and Dursun, 2008; Ohno et al., 2015). Parkinsonian symptoms usually occur in a few weeks after starting the antipsychotic treatment. Bradykinesia refers to reduced motor activity and slowing movements, which leads to akinesia in more severe cases. Tremor is an involuntary, rhythmic muscle contraction and relaxation (oscillation or twitching movements), affecting the hands, feet and head especially during resting state. In addition, affected patients often exhibit a stooped posture with increased muscle tone (rigidity) and a slow gait without arm swing.

### Akathisia

Patients with akathisia suffer from restlessness and repetitive movements of the legs and feet (Keefe et al., 2007; Haddad and Dursun, 2008). As a result, they cannot keep sitting and frequently shift their body position. Akathisia usually appears soon after starting antipsychotics or after increasing the dose.

### Dystonia

Dystonia causes sustained muscle contraction, often leading to postural distortion (Haddad and Dursun, 2008). Dystonia often attacks the neck muscles, tongue, trunk, and limbs. Acute dystonia usually appears in the first week after starting or increasing the dose of antipsychotics.

### Neural Mechanism of EPS Induction

**Figure 4:**
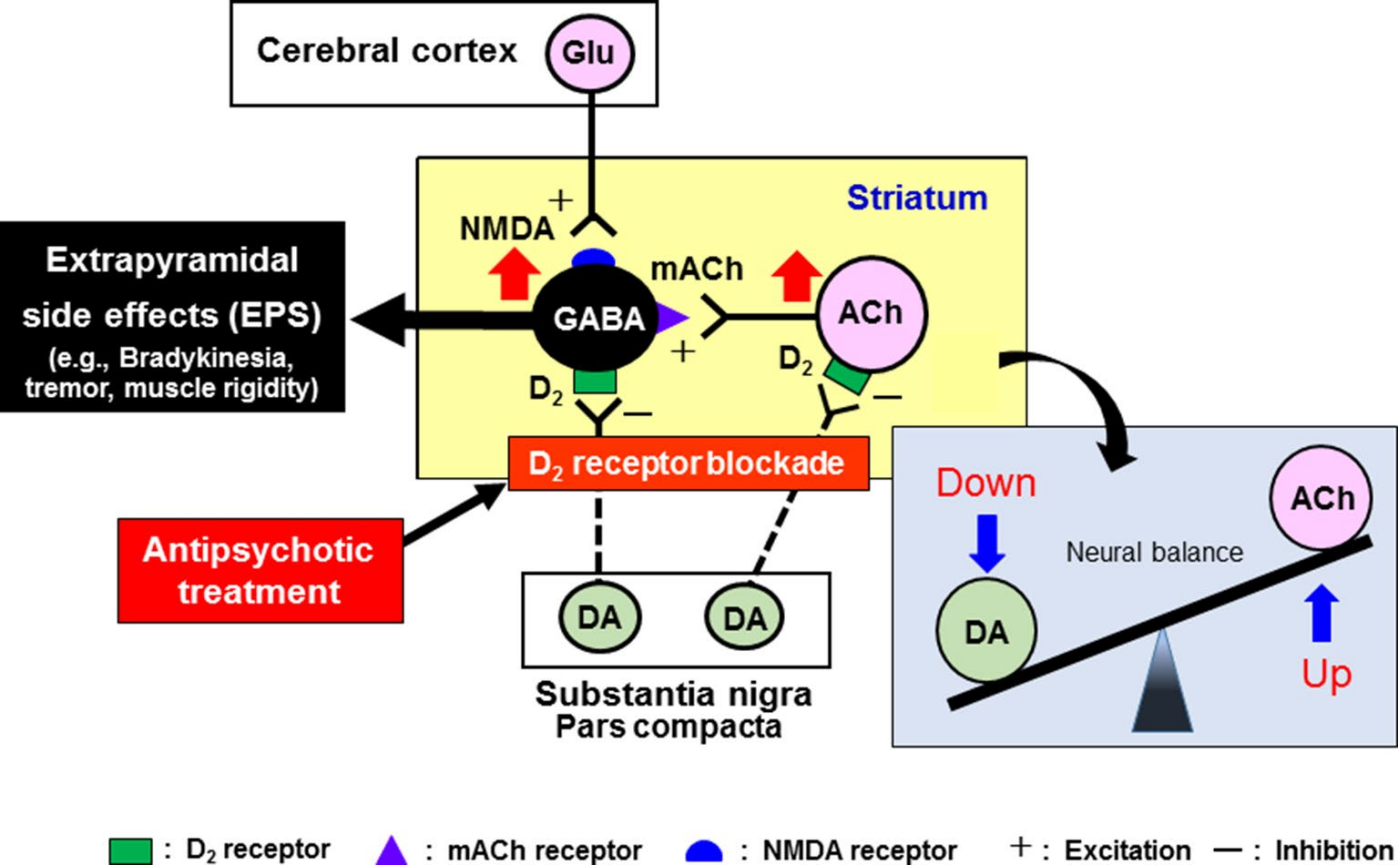
Pathophysiological mechanisms underlying the induction of extrapyramidal side effects (EPS) with antipsychotic treatments. Antipsychotic drugs commonly exert dopamine D_2_ blocking actions in the striatum, which relieves the striatal neurons (GABA-containing medium spiny neurons and acetylcholine (ACh)-containing interneurons) from negative regulation by the nigrostriatal dopaminergic neurons. Thus, overall activation of striatal medium spiny neurons by antipsychotics evokes EPS (e.g., bradykinesia, tremor, and muscle rigidity). Antipsychotic-induced EPS can be alleviated by anti-muscarinic drugs (e.g., trihexyphenidyl and biperidene), which reverses the imbalance between dopamine and ACh neuron activities in the striatum. However, due to the side effects, these agents are not recommended for the elderly patients.

It is well known that antipsychotic-induced EPS are caused by the blockade of dopamine D_2_ receptors in the striatum (caudate-putamen) (Ohno et al., 1997; Ohno et al., 2013; Ohno et al., 2015; Ohno, 2019) (**Figure 4**). The GABAergic medium spiny neurons in the striatum receive excitatory glutamatergic inputs from the cerebral cortex and acetylcholinergic inputs from striatal interneurons. The medium spiny neurons also receive inhibitory dopaminergic inputs from the substantia nigra pars compacta (SNc) and express a high density of D_2_ receptors (Ohno et al., 2015). In addition, the dopaminergic neurons from the SNc also negatively regulate activities of the acetylcholinergic interneuron *via* D_2_ receptors. Most antipsychotic drugs commonly act as dopamine D_2_ receptor antagonists and activate the medium spiny neurons and acetylcholinergic interneurons in the striatum, eliciting various EPS symptoms (Ohno et al., 2013) (**Figure 4**).

To reduce EPS, a series of atypical antipsychotics, that show potent 5-HT_2_ blocking activities have been developed in the last three decades (Ohno et al., 1997; Ohno et al., 2012) (**Figures 2** and **3**). These agents include risperidone, perospirone, olanzapine, quetiapine, lurasidone, and paliperidone, and they commonly exhibit higher 5-HT_2_ than D_2_ affinities. Since olanzapine and quetiapine also show high affinities for other multi-receptors (e.g., histamine H_1_, adrenergic α_1,_ and muscarinic acetylcholine (mACh) receptors), these drugs are sometimes called as MARTAs and distinguished from SDAs.

**Figure 5:**
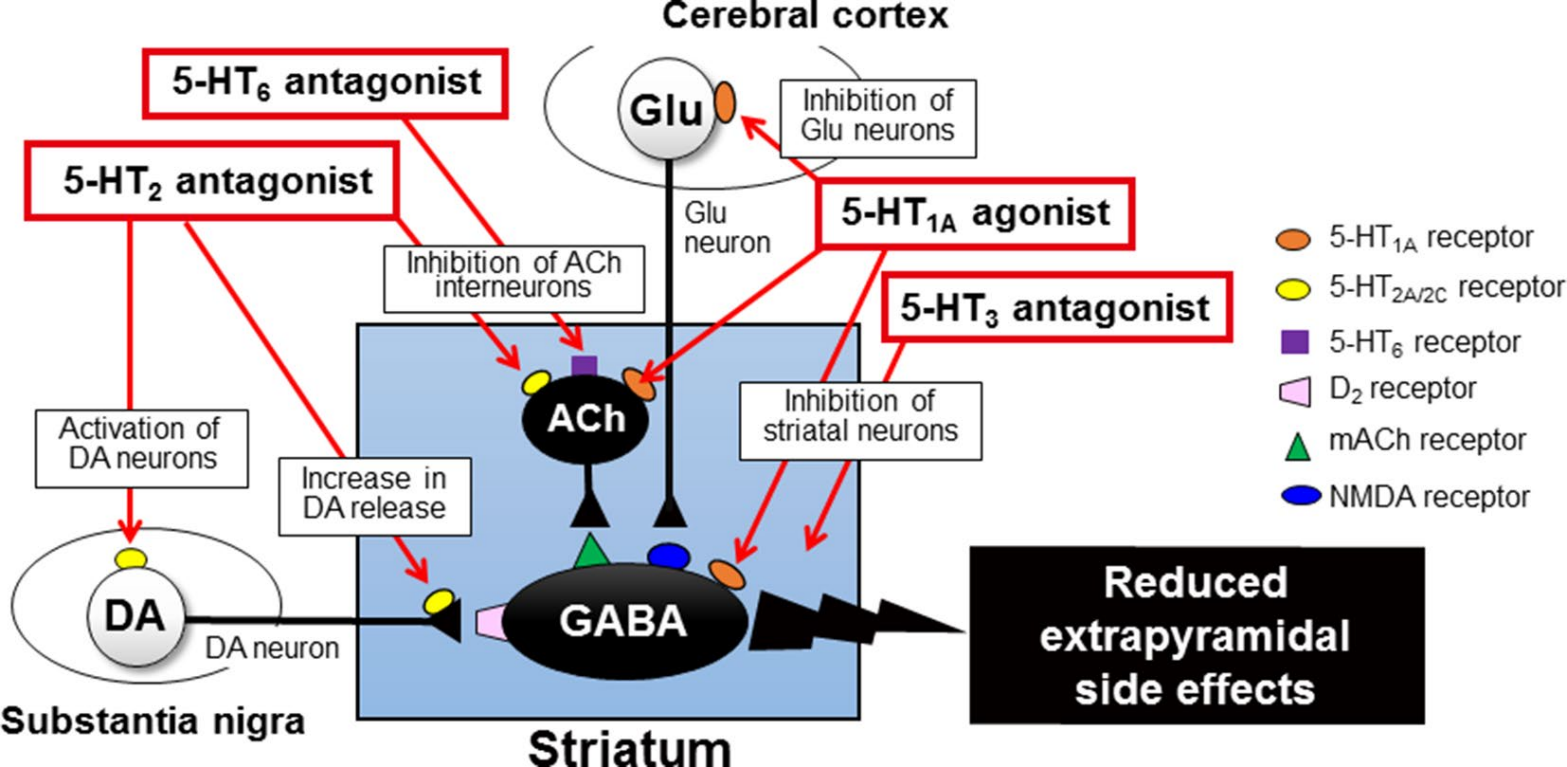
Mechanisms underlying serotonergic modulation of antipsychotic-induced extrapyramidal side effects (EPS). Activation of 5-HT_1A_ receptors, especially postsynaptic 5-HT_1A_ receptors in the striatum and cerebral cortex, alleviates antipsychotic-induced EPS. Blockade of 5-HT_2_ receptors on nigral dopamine neurons and their nerve terminals in the striatum can relieve the negative serotonergic regulation and thereby can increase the dopaminergic activities, which contributes to EPS reduction. Similarly, blockade of 5-HT_3_ and 5-HT_6_ receptors attenuates antipsychotic-induced EPS possibly *via* acting in the striatum. This figure is quoted and arranged from Biol. Pharm. Bull. 36, 1396, 2013.

It is well documented that blockade of 5-HT_2_ receptors attenuates antipsychotic-induced EPS associated with the striatal D_2_ receptor blockade (**Figure 2**). 5-HT_2_ receptors are located on nerve terminals and cell bodies of dopaminergic neurons in the striatum and the SNc, respectively, and inhibit dopaminergic neuron activities (Ohno et al., 1997; Ohno et al., 2012; Ohno et al., 2013). It is therefore proposed that blockade of 5-HT_2_ receptors relieves 5-HT_2_ receptor-mediated inhibition of dopamine release in the striatum and of dopamine neuron firing in the SNc, which leads to alleviation of EPS (**Figure 5**) (Remington and Kapur, 1999; Kapur and Remington, 2001). In fact, blockade of 5-HT_2_ receptors can reverse various responses of striatal neurons to antipsychotics (D_2_ receptor blockade), such as the enhancement of acetylcholine (ACh) release, the increase in metabolic turnover rate of dopamine and the induction of Fos protein expression, in the striatum Ohno et al., 1997; Ohno et al., 2013).

## Serotonergic Modulation of Antipsychotic-Induced EPS

As described previously, the serotonergic nervous system plays an important role in modulating EPS induction. Specifically, antipsychotic-induced EPS is augmented by stimulation of 5-HT_2_ receptors and attenuated by 5-HT_2_ receptor blockade. Besides 5-HT_2_ receptors, several 5-HT receptor subtypes, including 5-HT_1A_, 5-HT_3_ and 5-HT_6_ receptors, are involved in regulation of EPS induction associated with antipsychotic treatment (Ohno et al., 2013; Ohno et al., 2015).

5-HT_1A_ receptors function as both presynaptic autoreceptors and postsynaptic receptors, which inhibits neural activities *via* activating G-protein-gated inwardly rectifying K^+^ channels (Baumgarten and Grozdanovic, 1995; Barnes and Sharp, 1999; Shimizu et al., 2013a; Shimizu et al., 2013b; Ohno, 2019). Activation of 5-HT_1A_ receptors is known to reduce antipsychotic-induced EPS and motor disorders in animal models of Parkinson’s disease (Neal-Beliveau et al., 1993; Wadenberg et al., 1999; Mignon and Wolf, 2002; Ohno et al., 2008a; Ohno et al., 2008b; Ohno et al., 2009; Shimizu et al., 2010). Our previous studies showed that selective 5-HT_1A_ agonists (e.g., 8-OH-DPAT) ameliorated haloperidol-induced EPS (e.g., bradykinesia and catalepsy) and reversed the striatal Fos protein expression by the haloperidol treatment (Ohno et al., 2008a; Ohno et al., 2008b; Ohno et al., 2009). In addition, the anti-EPS action of 5-HT_1A_ agonists persisted against the denervation of 5-HT neurons with *p*-chlorophenylalanine treatment, illustrating that postsynaptic 5-HT_1A_ receptors are responsible for EPS reduction (Neal-Beliveau et al., 1993; Mignon and Wolf, 2002; Ohno et al., 2008a; Ohno et al., 2008b) Furthermore, microinjection of 5-HT_1A_ agonists into the striatum or the cerebral cortex (i.e., motor cortex) also attenuated extrapyramidal disorders (Shimizu et al., 2010). Therefore, it is likely that activation of 5-HT_1A_ receptors reduces antipsychotic-induced EPS by inhibiting neural activity in the striatum and motor cortex (**Figure 5**). Nonetheless, several studies suggest that presynaptic 5-HT_1A_ autoreceptors are also involved to reduce EPS (Wadenberg et al., 1999; Mombereau et al., 2017).

5-HT_3_ receptors function as cation (Na^+^, K^+^, and Ca^2+^)-permeable ion channels and excite target neurons (Barnes and Sharp, 1999; Ohno, 2019). Several studies demonstrated that blockade of 5-HT_3_ receptors reduced haloperidol-induced EPS (e.g., catalepsy and bradykinesia) (Silva et al., 1995; Ohno et al., 2011; Tatara et al., 2012) (**Figure 5**). Clinical studies also showed that the selective 5-HT_3_ antagonist, ondansetron, reduced the incidence and severity of antipsychotic-induced EPS in the schizophrenia treatment (Zhang et al., 2006; Akhondzadeh et al., 2009).

5-HT_6_ receptors are highly expressed in the basal ganglia (e.g., striatum), as well as the limbic (e.g., olfactory tubercles and hippocampus) and cortical regions (Barnes and Sharp, 1999; Ohno, 2019). We previously showed that the selective 5-HT_6_ antagonist, SB-258585, alleviated haloperidol-induced bradykinesia and catalepsy (Ohno et al., 2011; Tatara et al., 2012). In addition, EPS induction was also reduced by microinjection of SB-258585 into the striatum, implying that blockade of the striatal 5-HT_6_ receptors is at least partly involved in alleviating EPS. Since 5-HT_6_ receptors positively regulate the neural activities of the striatal ACh interneurons (Bonsi et al., 2007), it is conceivable that 5-HT_6_ antagonists reduce antipsychotic-induced EPS by inhibiting them (**Figure 5**).

Regarding other 5-HT receptor subtypes, neither 5-HT_4_ (GR-125487), 5-HT_5a_ (SB-699551), nor 5-HT_7_ (SB-269970) antagonists affected antipsychotic-induced EPS (Ohno et al., 2011). Therefore, the modulatory roles of these 5-HT receptors in modulating EPS appear to be minimal.

## Effects of Anti-Alzheimer’s Disease Drugs on Antipsychotic-Induced EPS

Alzheimer’s disease is the major component of elderly dementia. Since Alzheimer’s disease accompanies the loss of ACh neurons (Fibiger, 1991; Silva et al., 2014), several ChEIs such as donepezil, galantamine, and rivastigmine, which can increase the ACh level by inhibiting cholinesterase, are widely used to treat the cognitive impairment in Alzheimer’s disease. In addition, an NMDA receptor antagonist, memantine, is also used to alleviate the cognitive impairment. These anti-Alzheimer’s disease drugs are often prescribed in combination with antipsychotic drugs which can reduce BPSD (Salamone et al., 2001; Kozman et al., 2006), giving greater efficacy than monotherapy (Schmitt et al., 2004).

**Figure 6:**
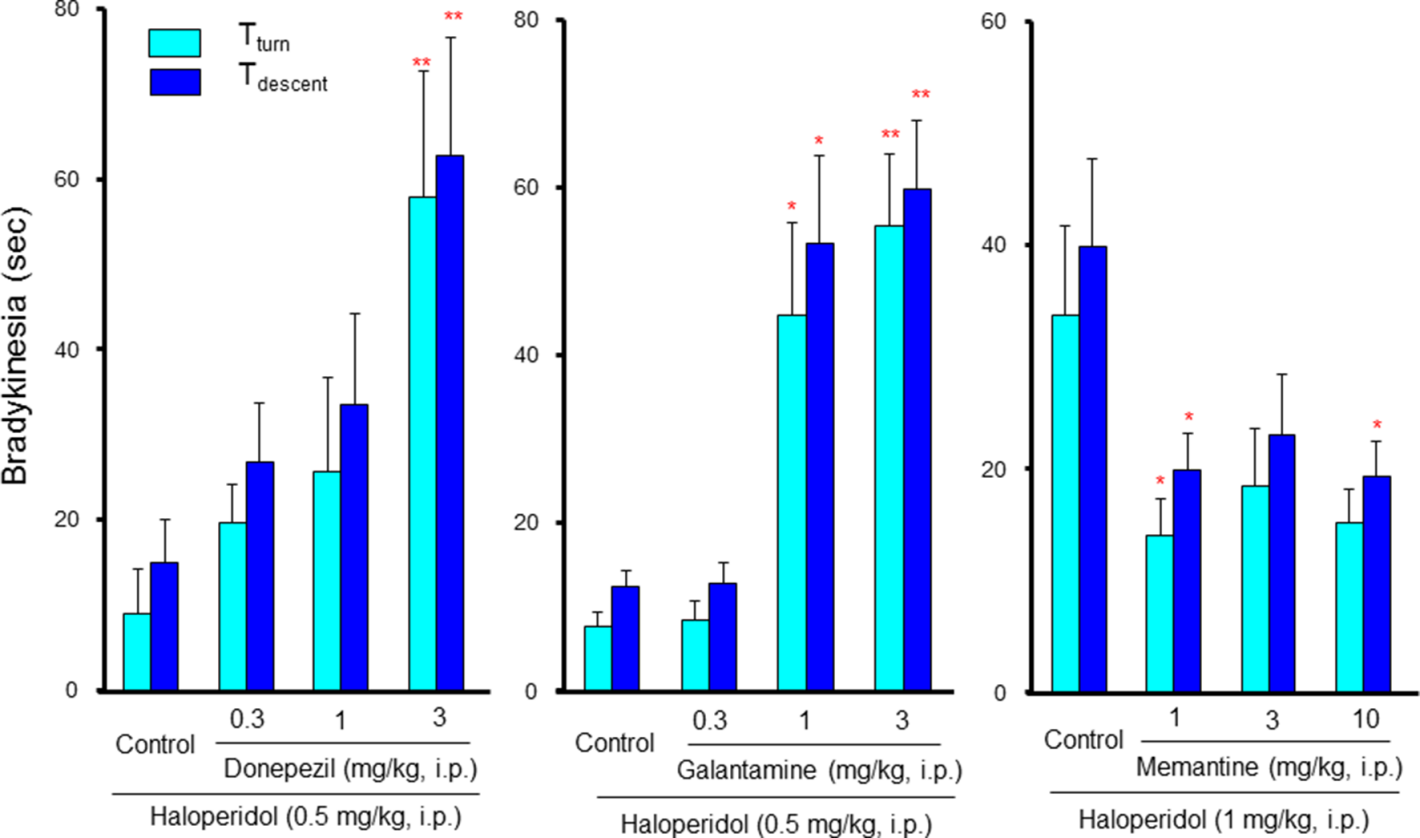
Interactions between anti-Alzheimer’s disease drugs and antipsychotics in induction of extrapyramidal side effects (EPS). Bradykinesia was estimated by the pole test, where mice were placed head-upward at the top of a pole (45 cm in height) and the time for mice to rotate downward (T_turn_) and to descend to the floor (T_descent_) was measured (Ohno et al., 2008a). Bradykinesia was evaluated as the prolongation of T_turn_ or T_descent_ Values. Although low dose (0.5 mg/kg) of haloperidol showed marginal effects in the pole test, combined treatment with cholinesterase inhibitors, donepezil, and galantamine, markedly potentiated haloperidol-induced bradykinesia in a synergistic manner. By contrast, the NMDA antagonist, memantine, significantly reduced bradykinesia induced by a high dose (1 mg/kg) of haloperidol. *P<0.05, **P,0.01; Significantly different from the control values. This figure is partly quoted and arranged from J. Pharmacol. Sci. 127, 439, 2015.

Although information on the drug interactions between antipsychotic and anti-Alzheimer’s disease drugs is limited, our previous study revealed that they markedly potentiated antipsychotic-induced EPS induction (Shimizu et al., 2015). Specifically, donepezil and galantamine rarely induce EPS signs when taken alone; however, they markedly potentiated bradykinesia induction by low dose of haloperidol in a dose-dependent and synergistic manner (**Figure 6**). In addition, the bradykinesia potentiation by galantamine was significantly reversed by a 5-HT_1A_ agonist (8-OH-DPAT), a 5-HT_2_ antagonist (ritanserin) and a 5-HT_6_ antagonist (SB-258585) (Shimizu et al., 2015). These findings indicate that caution is needed in the combined usage of antipsychotics and ChEIs in BPSD treatment. Furthermore, antipsychotics that can stimulate 5-HT_1A_ receptors or antagonize 5-HT_2_ and 5-HT_6_ receptors appear favorable as an adjunctive therapy for BPSD. Interestingly, in contrast to ChEIs, memantine, which antagonizes NMDA receptors, attenuated antipsychotic-induced EPS (**Figure 6**). Therefore, it seems likely that memantine is more favorable than ChEIs in the combined therapy of BPSD with antipsychotics.

Precise mechanisms underlying the synergistic potentiation of EPS by ChEIs is still unknown. However, the action of antipsychotics on cholinergic interneurons in the striatum seems to be involved since the firing of striatal cholinergic interneurons is negatively regulated by dopaminergic neurons and is reportedly facilitated by antipsychotics, increasing the ACh release (Damsma et al., 1990; DeBoer and Abercrombie, 1996). Therefore, ChEIs may augment the induction of EPS more potently in the presence of antipsychotics than their monotherapy.

## Effects of Antidepressant Drugs on Antipsychotic-Induced EPS

Antidepressant drugs, as well as antipsychotic drugs, are often used to treat BPSD, especially the mood disorders such as apathy, depression and emotional withdrawal (Lee et al., 2004; Trifirò et al., 2009; Brimelow et al., 2019; Sturm et al., 2018; Jin and Liu, 2019; Kales et al., 2019) (**Figure 6**). The majority of antidepressant drugs commonly inhibit neural reuptake of 5-HT and/or noradrenaline, and increase the synaptic levels of 5-HT and/or noradrenaline (Ohno, 2019). These drugs are generally classified as tricyclic antidepressants (TCAs) (e.g., nortriptyline, clomipramine, and imipramine), selective serotonin reuptake inhibitors (SSRIs) (e.g., fluoxetine, sertraline, and paroxetine) and serotonin noradrenaline reuptake inhibitors (SNRIs) (e.g., milnacipran, duloxetine, and venlafaxine). In addition, tetracyclic antidepressant drugs (e.g., mirtazapine and mianserin), which block adrenergic α2, 5-HT_2_ and 5-HT_3_ receptors without affecting 5-HT or noradrenaline transporters (Wood et al., 1993; Anttila and Leininen, 2001; Wikström et al., 2002; Fernández et al., 2005; Gillman, 2006), are also used to treat BPSD (**Figure 1**). These agents enhance noradrenaline and 5-HT release by inhibiting α2 autoreceptors on adrenergic nerve terminals and α2 heteroreceptors on serotonergic nerve terminals, respectively.

**Figure 7:**
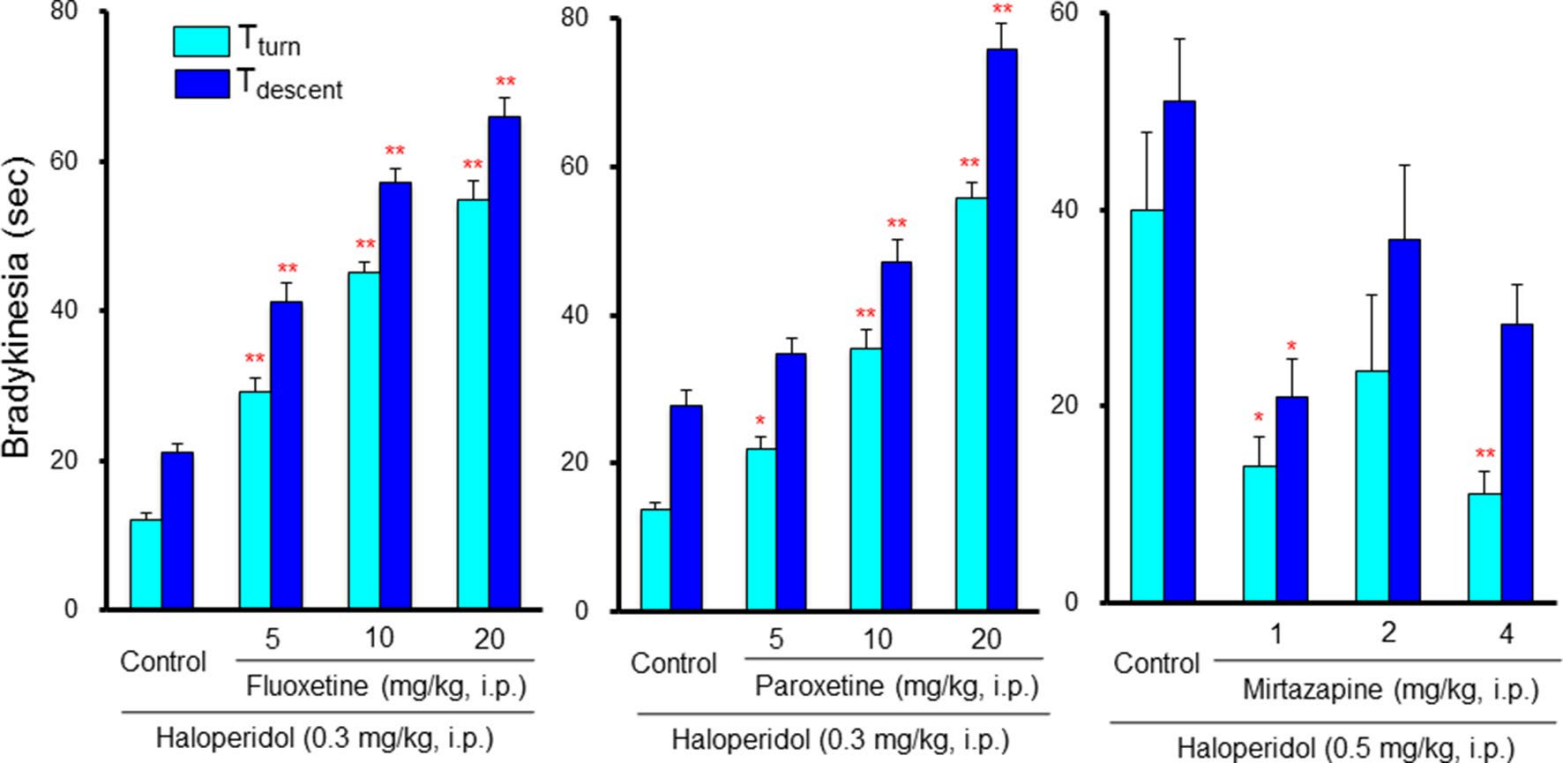
Interactions between antidepressants and antipsychotics in induction of extrapyramidal side effects (EPS). Bradykinesia was estimated by the pole test as described in **Figure 6** legend. Although low dose (0.3 mg/kg) of haloperidol showed only weak effects in the pole test, combined treatment with selective serotonin reuptake inhibitors, fluoxetine and paroxetine, markedly potentiated haloperidol-induced bradykinesia in a synergistic manner. By contrast, the tetracyclic antidepressant mirtazapine, which possesses α_2_, 5-HT_2_ and 5-HT_3_ antagonistic actions, reduced bradykinesia induced by a moderate dose (0.5 mg/kg) of haloperidol. *P<0.05, **P,0.01; Significantly different from the control values. This figure is quoted and arranged from Prog. Neuro-Psychopharmacol. Biol. Psychiatry 38, 252, 2012.

Neither SSRIs nor TCAs induced EPS by themselves; however, they markedly potentiated antipsychotic-induced bradykinesia and catalepsy in a dose-dependent manner (Tatara et al., 2012; Shimizu et al., 2013a; Shimizu et al., 2013b) (**Figure 7**). Clinical studies also showed that antidepressants worsen extrapyramidal motor disorders (Gill et al., 1997; Govoni et al., 2001; DeBattista and DeBattista, 2010). Therefore, caution should be taken in the combined usage of antidepressants with antipsychotics in BPSD treatment even though antidepressants do not cause EPS by themselves. Since both SSRIs and TCAs commonly enhance serotonergic activity, these agents potentiate antipsychotic-induced EPS probably by stimulating 5-HT_2_, 5-HT_3_ and 5-HT_6_ receptors. In addition, although the synergistic mechanism in potentiating EPS remains uncertain, antipsychotic-induced activation of striatal cholinergic interneurons may be involved since 5-HT excites the cholinergic neurons *via* 5-HT_2C_ and 5-HT_6_ receptors (Bonsi et al., 2007). In fact, blockade of 5-HT_2_ receptors by ritanserin, 5-HT_3_ receptors by ondansetron (5-HT_3_ antagonist), and 5-HT_6_ receptors by SB-258585 (5-HT_6_ antagonist), significantly attenuated the EPS augmentation by SSRIs (Tatara et al., 2012). In addition, stimulation of postsynaptic 5-HT_1A_ receptors by 8-HO-DPAT also alleviated SSRIs-induced EPS augmentation (Shimizu et al., 2013a; Shimizu et al., 2013b). This implies that antipsychotics which possess 5-HT_1A_ stimulating actions or 5-HT_2,_ 5-HT_3,_ and 5-HT_6_ blocking actions, could be useful as adjunctive therapies for BPSD.

In contrast to SSRIs and TCAs, tetracyclic antidepressants (mirtazapine and mianserin) did not augment, but rather attenuated antipsychotic-induced EPS (Tatara et al., 2012) (**Figure 7**). Thus, it seems likely that tetracyclic antidepressants are superior to SSRIs or TCAs in modulating EPS in combined treatment of BPSD with antipsychotics. Since the blockade of α_2_ receptors reportedly reduced antipsychotic-induced EPS (Imaki et al., 2009), EPS reduction by tetracyclic antidepressants is probably due to the α2 blocking action in addition to their 5-HT_2_ and 5-HT_3_ blocking activities.

## Drug Selection in BPSD Treatment

We reviewed antipsychotic use in BPSD treatment focusing on EPS, the most frequent side effects associated with the striatal D_2_ receptor blockade. Antipsychotic-induced EPS significantly disrupts activities of daily life and impairs the quality of life in the elderly patients with dementia. Therefore, information on the mechanisms and the drug interactions in modulating EPS induction are necessary to achieve proper pharmacotherapy of BPSD. In this regard, we should be very careful not only about EPS liability of antipsychotics by itself, but also about the interaction of antipsychotics with anti-Alzheimer’s disease drugs and antidepressant drugs.

Atypical antipsychotics (e.g., SDAs, MARTAs, and D_2_ partial agonists) are now the first line drug to treat psychosis and inappropriate behaviors (e.g., agitation and aggression) in patients with dementia. But, we should pay more attention to individual pharmacological characteristics of the atypical drug, especially their interactions with 5-HT receptor subtypes. Although most SDAs or MARTAs commonly possess high affinities to 5-HT_2_ receptors, many atypical antipsychotics shows a differential binding profile each other, interacting with various monoamine receptors (Farah, 2005). In fact, olanzapine additionally show high affinities for 5-HT_3_ and 5-HT_6_ receptors and acts as antagonist (Bymaster et al., 2001). In addition to 5-HT_2_ receptors, the SDA antagonist lurasidone also binds to 5-HT_1A_ receptors and acts as a partial agonist (Ishibashi et al., 2010). Furthermore, the dopamine D_2_ partial agonist aripiprazole also binds to 5-HT_1A_ and 5-HT_2_ receptors, and acts as a partial agonist and an antagonist, respectively (Stark et al., 2007). Since the actions of these agents with 5-HT receptor subtypes can reduce EPS caused by combined treatment of antipsychotics with anti-Alzheimer’s disease drugs and antidepressants, they could be a favorable BPSD treatment in terms of EPS management.

Among anti-Alzheimer’s disease drugs, the NMDA antagonist memantine appears superior to ChEIs in the combined BPSD therapy with antipsychotics as it attenuates antipsychotic-induced EPS. Likewise, the tetracyclic antidepressants (mirtazapine and mianserin) are recommended for combined use with antipsychotics to treat BPSD. Unlike 5-HT reuptake inhibitors (e.g., SSRIs, SNRI, and TCAs), these agents do not augment EPS induction, but alleviate antipsychotic-induced EPS, which is possibly by blocking α_2_, 5-HT_2_ and 5-HT_3_ receptors (Imaki et al., 2009; Ohno et al., 2011).

## Closing Remarks

This article provides information on the safe usage of antipsychotics in adjunctive therapy for BPSD in patients with dementia. The crucial roles of 5-HT receptors, especially 5-HT_1A_, 5-HT_2_, 5-HT_3_, and 5-HT_6_ receptors, in modulating antipsychotic-induced EPS were revealed. Although antipsychotic drugs are effective for psychosis, agitation, excitation, and abnormal behaviors, we should be very careful about drug selection in the combined use of antipsychotics with anti-Alzheimer’s disease drugs or antidepressants. Specifically, ChEIs and 5-HT reuptake inhibitors (SSRIs, SNRI, and TCAs) markedly potentiate antipsychotic-induced EPS in a synergistic manner. In contrast, the NMDA antagonist (memantine) or the tetracyclic antidepressants (mirtazapine and mianserin) seem to be more suitable for adjunctive therapy of cognitive impairment and mood disorders of BPSD, respectively. Furthermore, antipsychotics which have 5-HT_1A_ agonistic actions or 5-HT_2_, 5-HT_3,_ and 5-HT_6_ antagonistic actions appear to be useful for adjunctive BPSD treatment.

## Author Contributions

YO drafted the initial manuscript. All authors (YO, NK, SS) improved, contributed to and agreed on the final version of the manuscript.

## Funding

This study was partly supported by a research grant from by a Grant-in-Aid for Scientific Research from the Ministry of Education, Culture, Sports, Science and Technology (YO:17K08324, SS:16K21501) and from the Smoking Research Foundation (YO).

## Conflict of Interest Statement

The authors declare that the research was conducted in the absence of any commercial or financial relationships that could be construed as a potential conflict of interest.
